# PRETUS: A plug-in based platform for real-time ultrasound imaging research

**DOI:** 10.1016/j.softx.2021.100959

**Published:** 2022-01

**Authors:** Alberto Gomez, Veronika A. Zimmer, Gavin Wheeler, Nicolas Toussaint, Shujie Deng, Robert Wright, Emily Skelton, Jackie Matthew, Bernhard Kainz, Jo Hajnal, Julia Schnabel

**Affiliations:** a School of Biomedical Engineering & Imaging Sciences, King’s College London, UK; b Department of Informatics, Technical University Munich, Germany; c Department of Computing, Imperial College London, UK; d Friedrich-Alexander-University Erlangen-Nürnberg, Germany; e Helmholtz Zentrum München – German Research Center for Environmental Health, Germany

**Keywords:** Real time, Ultrasound imaging, Plug-in based

## Abstract

We present PRETUS — a Plugin-based Real Time UltraSound software platform for live ultrasound image analysis and operator support. The software is lightweight; functionality is brought in via independent plug-ins that can be arranged in sequence. The software allows to capture the real-time stream of ultrasound images from virtually any ultrasound machine, applies computational methods and visualizes the results on-the-fly.

Plug-ins can run concurrently without blocking each other. They can be implemented in C++ and Python. A graphical user interface can be implemented for each plug-in, and presented to the user in a compact way. The software is free and open source, and allows for rapid prototyping and testing of real-time ultrasound imaging methods in a manufacturer-agnostic fashion. The software is provided with input, output and processing plug-ins, as well as with tutorials to illustrate how to develop new plug-ins for PRETUS.

## 1 Motivation and significance

Ultrasound (US) imaging is one of the most widely used medical imaging modalities, because it is portable, affordable and safe, and can be used to gain insight about most body organs. Moreover, US is, unlike other common modalities such as Computed Tomography (CT), Magnetic Resonance Imaging (MRI) or X-ray, a real-time modality by design: to use an ultrasound system the operator needs to interpret the real-time stream of images shown on the display and use the interpreted information to guide the US transducer to the desired view. As the examination progresses, the operator typically stores a few tens of static images or short clips for reporting or further investigation. Importantly, the main clinical use of US images is during the procedure. This is because in diagnostic imaging, diagnosis is done by the operator as the images are being acquired and interpreted. In interventional imaging, surgical tools are guided using images in real-time image.

US image analysis is a very active area of research [1–3], and most published work has focused in the ‘offline’ analysis of images and clips stored by the operator as described above. However, real-time analysis of US image streams can potentially transform the way ultrasound is utilized since it can provide the operator with extended information and guidance *during* the examination, which as pointed out before offers the biggest potential benefit.

We identified three main reasons why limited work has been done on real-time US image analysis: firstly, collecting real-time data is not supported by most US systems and requires external equipment, such as a video framegrabber; the few systems which do support real-time streaming of DICOM (Digital Imaging and Communications in Medicine, an international standard for storage and transmission of medical images — https://www.dicomstandard.org) frames require a proprietary protocol to access the image stream. Secondly, existing research tools used for implementing real-time image analysis methods are designed to perform a single computational task on the stream of images, however real-time analysis often requires a number of tasks to run in succession (or in parallel) without compromising the realtime performance. And thirdly, in order to carry out translational research, the results of the real-time analysis must be shown to the operator in a way that does not require switching between displays during the scan, as this would be unfeasible. In this paper we describe *PRETUS: Plugin-based Real Time UltraSound*, a software that addresses these three challenges while remaining a simple, lightweight tool that can be easily extended via plug-ins — independent pieces of software that can be built separately to the main software and can be added dynamically to extend its functionality.

A number of research softwares have been proposed over the last years supporting real-time ultrasound imaging for research purposes. Of those, the most widely used are Slicer IGT [4] and MITK IGT [5]. Slicer IGT was one of the first software tools to enable easy implementation of image guided intervention software, by integrating existing navigation tools (e.g. the PLUS toolkit — www.plustoolkit.org, and OpenIGTLink [6]) into Slicer (www.slicer.org), a general-purpose medical imaging software written in C++. Slicer IGT is designed as a layer on top of PLUS (which connects and manages data from sensors and image sources) providing a wide, extensible collection of algorithms, and on top of which an application specific GUI and logic can be built. Conveniently, Slicer’s functionality can be extended by custom Python-scripted modules. MITK IGT was published later, and followed a similar paradigm: incorporate image guided tools into MITK, a general purpose image processing software. MITK does have a Python module that allows to query data using Python commands.

As opposed to Slicer IGT and MITK IGT, PRETUS is a minimal software that has no functionality on its own, other than connecting plug-ins and ensuring that they can run concurrently and communicate between them. All the functionality is brought in by plug-ins, that are built as dynamic libraries loaded at runtime. This facilitates a crucial paradigm shift with respect to MITK IGT or Slicer IGT: instead of aiming at being compatible with the greatest number of devices, PRETUS is conceived to be as independent as possible from specific devices, by delegating most functionality to plug-ins, so that if required a self-contained device specific plug-in can be implemented. This design paradigm also promotes that functionality is modular, and that each plug-in does a simple task on a specific input and produces a specific output. Additionally, this allows a very flexible interconnection of plug-ins, for example enabling multiple inputs, outputs, and plugins interconnected in arbitrary ways as defined by the user that can be changed during the imaging session. Last, a major difference is that PRETUS plug-ins run synchronously at a user-defined frame rate, and not when data is available. As shown later, this has the advantage of enabling overall real time behavior even in conditions where plug-ins have a slow execution by trading-off frame rate. These differences is summarized in Table 1.

PRETUS was developed within the iFIND project (www.ifindproject.com) to collect data and test methods in over 500 pregnant patients. The software has been used for 2D and 3D ultrasound applications. Methods that have used PRETUS in 3D imaging applications include 3D whole-fetus imaging by fast registration of a sequence of 3D ultrasound volumes in real time [7,8], full placenta imaging by fusion and segmentation of the placenta from multiple 3D ultrasound views [9,10], and whole fetal head imaging using atlas-registration and fusion [11,12]. PRETUS has also been used in real time 2D applications, such as standard fetal plane detection [13], automatic biometric measurements in standard fetal planes [14,15], and automatic detection and localization of fetal organs from ultrasound images [16]. PRETUS is also being used in research towards implementing AI-enabled ultrasound methods in low and middle income countries in the context of the VITAL project (http://vital.oucru.org/), specifically for lung ultrasound in dengue patients [17].

The software is used via a command-line executable where the user defines a real-time pipeline at run time. The specific experimental setting will depend on the desired pipeline, however a typical setting would be to define an input imaging source (e.g. a file from disk or a framegrabber), a processing task (for example, detecting standard planes) and an output task (e.g. display the results on a screen). More examples and use cases are described in more detail in Section 3.

In summary, PRETUS is a lightweight, extensible software that addresses the three challenges outlined above as follows: firstly, by enabling the collection of real-time US data from virtually any machine using the video output. Secondly, by enabling realtime pipelines of multiple image processing and visualization steps concurrently. And thirdly, by showing both the live imaging stream, information and outputs from the different processing tasks in a live, compact and unified way. Moreover, PRETUS can take pre-recorded videos or images and play them back at acquisition frame-rate to simulate live sessions in the lab.

## 2 Software description

PRETUS is a command-line software, built using open-source software and tested in Linux (all dependencies are cross-platform, but limited testing has been carried out in Windows and Mac) to facilitate research on real-time ultrasound imaging. The software works by establishing a real-time processing pipeline with an arbitrary number of elements (plug-ins). The plug-ins in the pipeline carry out specific functions such as to generate a realtime stream of data (for example from an ultrasound video source), to apply real-time algorithms on the data stream (e.g. implemented via deep neural networks), and to output the result (for example by visualizing the processed images, or the metadata, or saving both to a file).

### 2.1 Software architecture

**Fig. 1:**
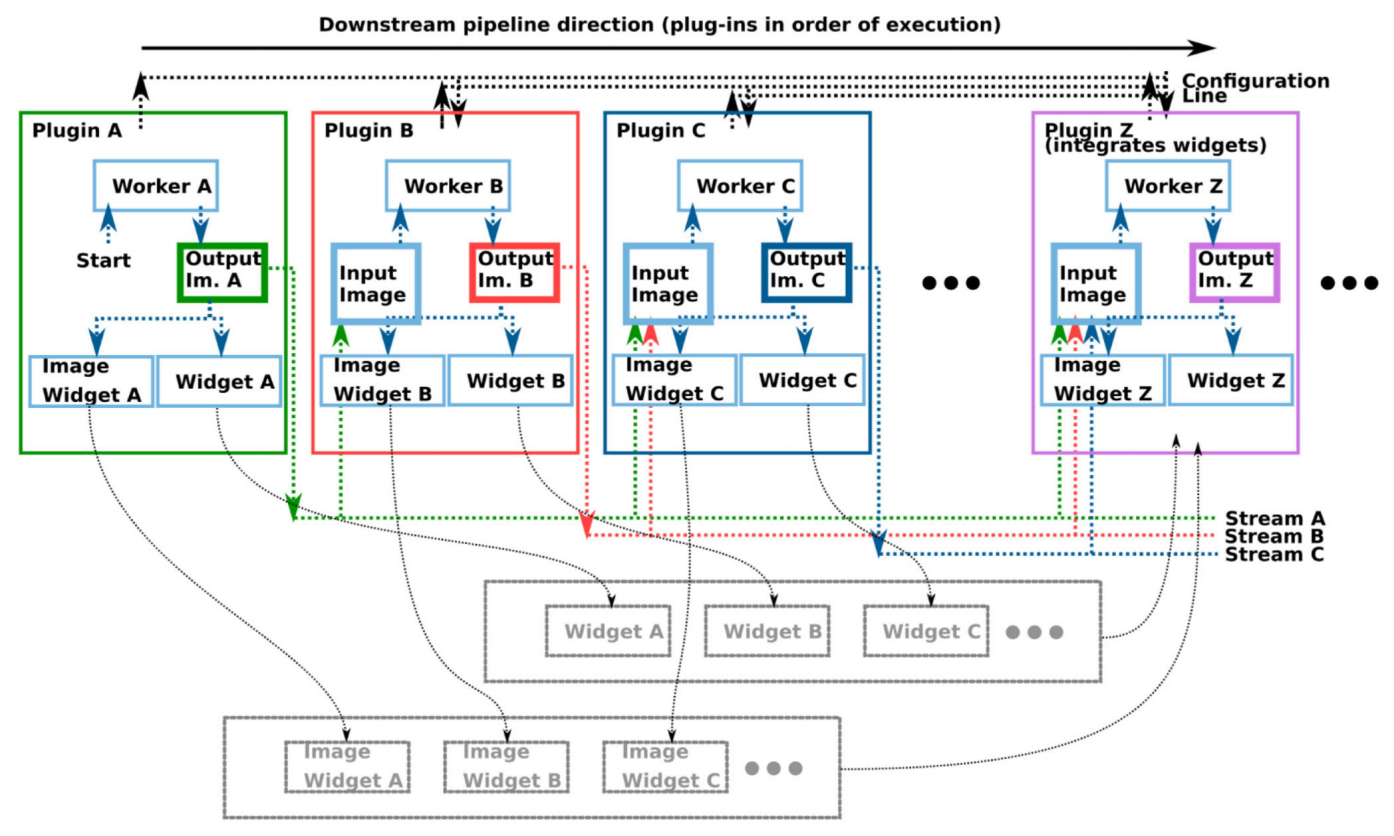
Overview of the plug-in pipeline concept. Each box represents a plug-in, inserted into the execution pipeline in order from left to right. The first plug-in (A) will normally be the imaging source (e.g. frame grabber or file reader) and the last plug-in will normally be for visualization (as illustrated here, integrating widgets from all plug-ins). Configuration is transmitted downstream (illustrated by the configuration line on top) and data is transmitted from each plug-in downstream using the Streams concept (Section 2.2.2).

The software is implemented as a lightweight QApplication (https://www.qt.io/), which interconnects and starts a number of plug-ins in a pipeline. Fig. 1 shows an illustration of the pipeline with the plug-ins and the data transmission lines, or data *Streams* described later in Section 2.2.2.

The software is organized into four modules:

- The PRETUS app, in the App folder. The application first loads and instantiates the plug-ins (implemented as dynamic libraries) that are found in the plug-ins folder (or folders) at run-time, also passing any command-line arguments to every plug-in. All plug-ins inherit from the Plugin class, which implements asynchronous callbacks (using QT signals and slots) to transmit configuration information and data between plug-ins. These transmission lines (here referred to as *Streams* and described in more detail in Section 2.2.2) are established, and finally the plug-ins are *activated*, starting the execution loop for the entire plug-in pipeline.
- The Common module, which includes common classes, inherited from the iFIND project, to manage data. The main class in this module is the ifind::Image class which is used in PRETUS to encapsulate both images and metadata, and is transmitted through the signal/slots.
- The PluginLib library, which implements all classes that plug-ins need to inherit (mainly Plugin, Worker and Qt-PluginWidgetBase). This library is described in more detail in Section 2.2.1.
- The Plugins folder, which includes some basic plug-ins readily released with the software. The plug-ins included with this release are further described through examples in Section 3 and in Appendix A.

Since the main functionality is brought in via the plug-ins, in the following we describe the architecture of the plug-ins in more detail.

### 2.2 Software functionalities

PRETUS defines, at run time, a pipeline of imaging plug-ins that in sequence process a stream of images. The sources of the imaging data, the processes themselves and whether the outcomes are displayed and/ or stored depends on the plug-ins used. In terms of performance, PRETUS is designed to satisfy two main requirements. First, plug-ins can be executed concurrently, i.e., the work of each plug-in runs in a separate non-blocking thread. Second, plug-ins must run as close as possible to realtime. The work of each plug-in runs at a user-defined frame rate, and the latest available frame is processed when a previous computations has been completed. To this end, PRETUS will drop frames at the input of a plug-in until the plug-in is ready to accept a new one. This behavior can be overridden if a specific plug-in does not need real-time performance and processing of all frames is sought.

The two above functionalities are implemented through two mechanisms: the plug-in system, and the *Streams*.

#### 2.2.1 Plug-in system

Plug-ins are independent programs, built as dynamic libraries, and are loaded into PRETUS at run time. All plug-ins take images as input, yield images at the output, and normally delegate the processing task to a Worker class. Plug-ins can also have two complementary means of displaying information and outputs: (i) by implementing a widget that will typically show graphs, numbers and text, and allow for input through sliders and other widgets; and (ii) by implementing an image widget that will display images, overlays, masks, etc. Both the widget and the image widget must inherit from the QtPluginWidgetBase class in PluginLib. Two examples of how to build plug-ins for PRETUS are outlined in Section 3 and detailed in the repository (https://github.com/gomezalberto/pretus).

The basic operation of a plug-in in the pipeline is as follows:

1. The plug-in receives an input image from previous plugins in the pipeline. The image is also passed on to the next plug-in.
2. If the image belongs to the Stream(s) that this plug-in accepts, the image is sent to the plug-in’s timer.
3. If the Worker is not processing the previous image, the timer sends the latest image to the worker in a separate thread.
4. The main processing task of the plug-in is carried out in the Worker. When finished, the output image is sent to the plug-in and the timer is notified that the worker is ready to take a new image.
5. The plug-in sends the output image through the plug-in’s output Stream. Downstream plug-ins are now able to use it.
6. If the plug-in has a Widget and/or an ImageWidget, the output image is sent to them for display. The user can act on any inputs available in the widget (e.g. sliders, checkboxes, etc.) to make changes in the plug-in behavior during the imaging session.

We recommend (as we do in all plug-ins included in this repository) that any output image resulting from a plug-in’s task is added as a layer to the input image. Because images are transmitted as pointers, no data will be duplicated in memory, so this mechanism is efficient. Additionally, this allows to always track what image was used to produce what result, even if different Streams operate at different rates and while maintaining real-time performance.

#### 2.2.2 Streams

In this context, a *Stream* refers to every image sequence produced by a plug-in and accessible to all other downstream plugins in the pipeline. Every stream is named after the plug-in that generates it, with the exception of the plug-ins that generate data at the source, also called input plug-ins.

Input plug-ins capture imaging data and transmit it down-stream the pipeline. In the current release of PRETUS, three input plug-ins are provided: the Video Manager plug-in, that can read and transmit frames from a video file; the Frame Grabber plug-in, that reads video output from an ultrasound system and transmits it frame by frame; and the File Manager, that reads images from a folder system and transmits them at a given framerate. These type of plug-ins must return true via the IsInput() plug-in method. The Stream transmitted by an input plug-in is called ‘Input’ regardless of the plug-in name. Multiple input plug-ins can be used simultaneously, in which case only the first will have an Stream called ‘Input’, and the rest (in order of appearance in the pipeline) will be called ‘Input1’, ‘Input2’, etc. The rest of the plug-ins in the pipeline will by default accept images from ‘Input’ but can be set to use other inputs with the command-line option --<pluginname>_stream Input1 or using the menu in the widget during the imaging session.

#### 2.2.3 Building a plug-in

Plug-ins are dynamic libraries written in C++, that link against the Plugin library provided with PRETUS. The processing task carried out by the plug-in can be implemented in C++, or in Python. To illustrate the two types of plug-ins, the repository includes two sample plug-ins in the Plugins folder designed and documented to serve as templates and tutorials for developers to implement their C++plug-ins (Plugin_CppAlgorithm) and their Python plug-ins (Plugin_PythonAlgorithm).

### 2.3 Hardware requirements and experimental configuration

RETUS has been built and tested in a basic laptop, which defines the minimum recommended requirements and set a lower bound for performance. Such performance is quantified on exemplar plug-ins in Section 3.3.

The laptop was a DELL XPS 15 9550 from 2015, with 16 GB RAM, a Intel Core i7-6700HQ CPU @ 2.60 GHz × 8 CPU, and a NVIDIA GeForce GTX 960M 2 GB GPU. The laptop was configured with Ubuntu 20.04 LTS.

## 3 Illustrative examples

**Fig. 2:**
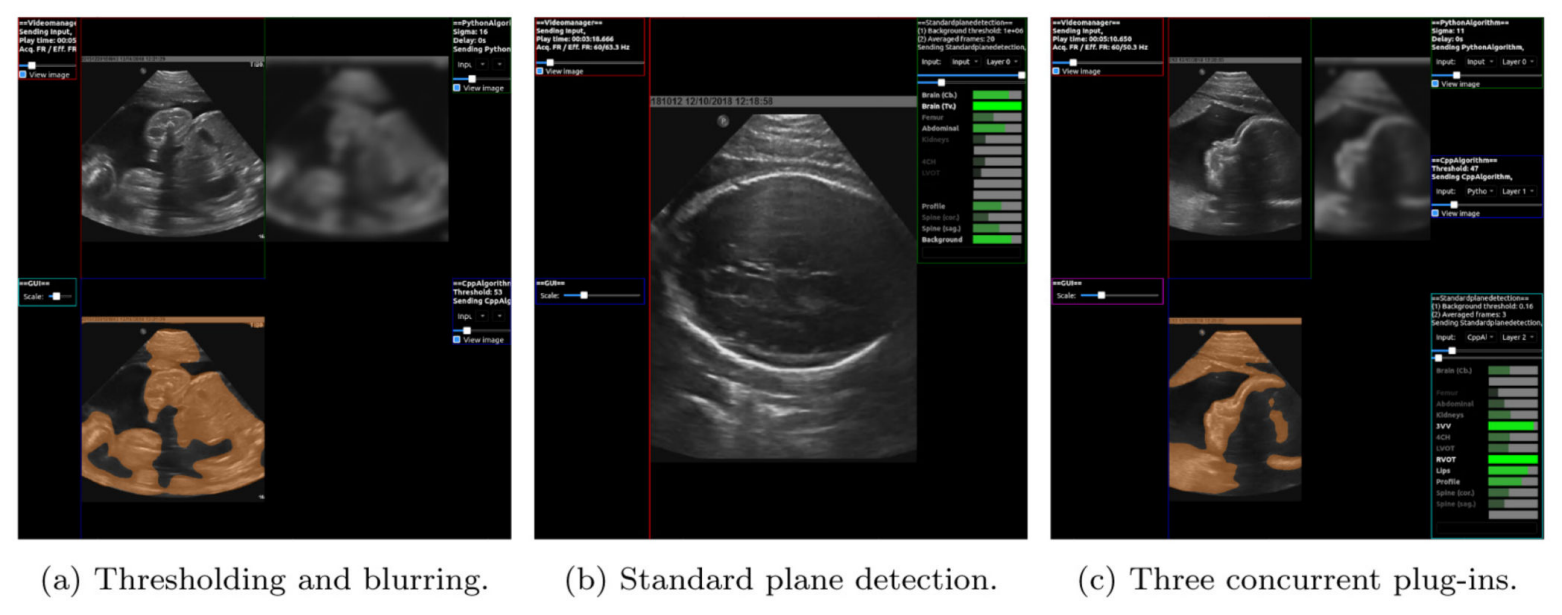
Screenshot of three PRETUS pipelines. (a) pipeline where the input image is shown in the top left, the blurred image on the top right, and the thresholded version of the blurred image, overlaid onto the input, on the bottom left. (b) capture of the Standard Plane Detection plug-in integrating SonoNet in PRETUS where a ‘Head’ standard view has been detected. (c) three plug-ins (blurring, thresholding and Sononet) working concurrently. All examples are described in Section 3, and illustrated in the videos in the supplementary material.

In this section we describe three usage examples: first, an example of real-time blurring and thresholding of an ultrasound video. These plug-ins are not designed with an intended application in mind other than exemplifying the design and performance of PRETUS. Second, an example showing the integration of SonoNet [13], a deep neural network for the automatic identification of anatomical fetal standard view planes, into PRETUS. And third, blurring, thresholding and SonoNet working in the same pipeline, where we evaluate the real-time performance with concurrent C++and Python plugins. In all cases the videos were 15 min long and were encoded at 30 frames per second. Videos showing the three examples in action is provided in the supplementary material, and a screen shot of these videos is shown in Fig. 2.

### 3.1 Blurring and thresholding

The purpose of this example is to illustrate the connection of the output of a plug-in to the input of another plug-in, and the visualization of the results. To this end, we build a pipeline with four plugins: the video manager plug-in (Appendix A.2) as the input plug-in, which reads a video from file and transmits each frame through the pipeline. Then, the Python Algorithm plug-in (Appendix A.5) takes the video frames and applies a blurring operation. The blurred frame is input to the Cpp Algorithm plug-in (Appendix A.4), which performs a binary thresholding operation on the blurred image. Finally, the GUI plug-in (Appendix A.8) takes all widgets from the three previous plug-ins and displays them on screen.

All plug-ins accept, by default, images from the Input stream. This stream is generated by any of the input plug-ins (video manager, frame grabber and file manager) which are first in the pipeline. For the Cpp Algorithm plug-in to receive the output of the Python Algorithm plug-in as input, we use the optional argument --cppalgorithm_stream pythonalgorithm (or select the input from the Python Algorithm, and the last layer, in the widget). In addition, the Python Algorithm adds the blurred image as an additional layer to its input image, and the Cpp Algorithm plug-in needs to be informed of what layer from the pythonalgorithm stream to use, in this case the last one, with the command line argument --cppalgorithm_layer -1. The complete command line call for this example is: $ ./bin/pretus -pipeline "videomanager>pythonalgorithm>cppalgorithm>gui"
  −−videomanager_input ~/data/video.MP4 \
  −−cppalgorithm_stream pythonalgorithm \
  −−cppalgorithm_layer -1

The program will launch, display information about the plug-ins used as below, and open a window with the GUI (Fig. 2, left, and Video SV1). Loading plug-ins from <folder>
  0 [Plugin] loading <folder>/libPlugin_filemanager.so...      File manager(0) loaded
  1 [Plugin] loading <folder>/libPlugin_imageFileWriter.so...  Image file writer(1) loaded
  2 [Plugin] loading <folder>/libPlugin_videomanager.so...     Video manager(2) loaded
  3 [Plugin] loading <folder>/libPlugin_CppAlgorithm.so...     Cpp Algorithm(3) loaded
  4 [Plugin] loading <folder>/libPlugin_framegrabber.so...     Frame grabber(4) loaded
  5 [Plugin] loading <folder>/libPlugin_PythonAlgorithm.so...  Python Algorithm(5) loaded
  6 [Plugin] loading <folder>/libPlugin_planeDetection.so...   Standard plane detection(6) loaded
  7 [Plugin] loading <folder>/libPlugin_GUI.so...     UI(7)   loaded
Video manager -> Python Algorithm
Python Algorithm -> Cpp Algorithm
Cpp Algorithm -> GUI
VideoManager::Initialize() - loading video ~/data/video.MP4... loaded, FPS = 60, frames = 110842
Start acquisition
Manager::exitLoop() - Enter ‘quit’ to exit:
>>

The program will exit by entering ‘quit’ in the command line.

### 3.2 SonoNet Integration

In this example we illustrate the integration of SonoNet [13], a model to detect standard fetal planes for the 20 week fetal screening ultrasound examination. The SonoNet model is incorporated into pretus via the Standard Plane Detection plug-in.

Since SonoNet is implemented in a frame by frame basis, in our implementation we allow the user to use a temporal average to leverage high acquisition frame rates to stabilize the plane prediction. The number of frames to be averaged can be set by command line argument and modified in real time via a slider in the widget. With this, the resulting call is: $ ./bin/pretus -pipeline “videomanager>standardplanedetection>gui” \
  −−videomanager_input ~/data/video.MP4 --standardplanedetection_taverage 20

The resulting display and interactions can be seen in Fig. 2, middle, and Video SV2.

### 3.3 All plug-ins in the same pipeline

The purpose of this example is to demonstrate that multiple C++and Python plug-ins can work concurrently, and to evaluate to the effect of delay and execution time of each plug-in in the performance of the entire pipeline and the overall delay with respect to the input stream. We use the Video Manager plug-in as input, and use the Python Algorithm, the Cpp Algorithm and SonoNet in the pipeline, followed by the GUI plug-in. To illustrate the behavior of frame-dropping at high delays, we introduce an artificial variable wait time in the Python Algorithm plug-in. All plug-ins are provided with the command-line option <pluginname>_time 1, which measures the execution time of the worker. We run the pipeline in five configurations: 1) with all plug-ins using the Input stream; and 2) to 5), with plugins connected in sequence, using as input the output stream of the previous plug-in, and a plug-in frame rate (identical for all three plug-ins) of 10 Hz (configuration 2), 20 Hz (configuration 3), 30 Hz (configuration 4), and 40 Hz (configuration 5). We also measure execution times for all plug-in. The execution call for the first configuration is: $ ./bin/pretus -pipeline “ideomanager>pythonalgorithm>cppalgorithm>standardpanedetection>gui” \
−−videomanager_input ~/data/video.MP4 \
−−standardplanedetection_time 1 --pythonalgorithm_time 1 --cppalgorithm_time 1 \
 −−pythonalgorithm_delay 0.1

For configurations 2) to 5) (replacing the frame rate value): $ ./bin/pretus -pipeline "videomanager<pythonalgorithm>cppalgorithm>standardpanedetection>gui" \
−−videomanager_input ~/data/video.MP4 --videomanager_verbose 1 \
−−standardplanedetection_time 1 --pythonalgorithm_time 1 --cppalgorithm_time 1 \
−−pythonalgorithm_framerate 10 --pythonalgorithm_delay 0.1 \
−−cppalgorithm_framerate 10 --standardplanedetection_framerate 10 \
−−cppalgorithm_stream pythonalgorithm --cppalgorithm_layer -1 \
−−standardplanedetection_stream cppalgorithm --standardplanedetection_layer -1

An example showing the visualization for configuration 2 can be seen in Fig. 2, right, and in Video SV3. The Table 2 shows the average ± standard deviation execution time per plug-in, for different wait times introduced in the first plug-in in the sequence (the Python Algorithm plug-in — PA). The statistics were computed over all calls to the plug-ins over the 15 min of the video. The video transmitted images at 30 Hz, yielding 27000 frames in total. Depending on the frame rate at which each plug-in was set, this results in between 3500 to 35000 independent time measurements for each plug-in. We conducted an n-way ANOVA test for the execution times reported by each plug-in and found that: for the Python Algorithm all execution times are significantly different (*p* < 0.01), as expected; for the Cpp Algorithm, only the first experiment (wait = 0 ms) is significantly different to the last two (150 and 200 ms), which are not different to each other (*p* < 0.01); and for the Standard plane detection, the first three experiments (0, 50 and 100 ms) and the last two (150 and 200 ms) are not significantly different within the groups but they are different between the groups (*p* < 0.01). In summary, and as the table shows, the wait times (from 0 to 200 ms) have very little effect in the execution time of other plug-ins downstream because each is executed on a separate thread. Interestingly, the effect is that with longer wait times the execution is slightly faster, possibly because since the plug-in waits but does not consume any CPU resource other plug-ins have more resources to themselves.

**Fig. 3:**
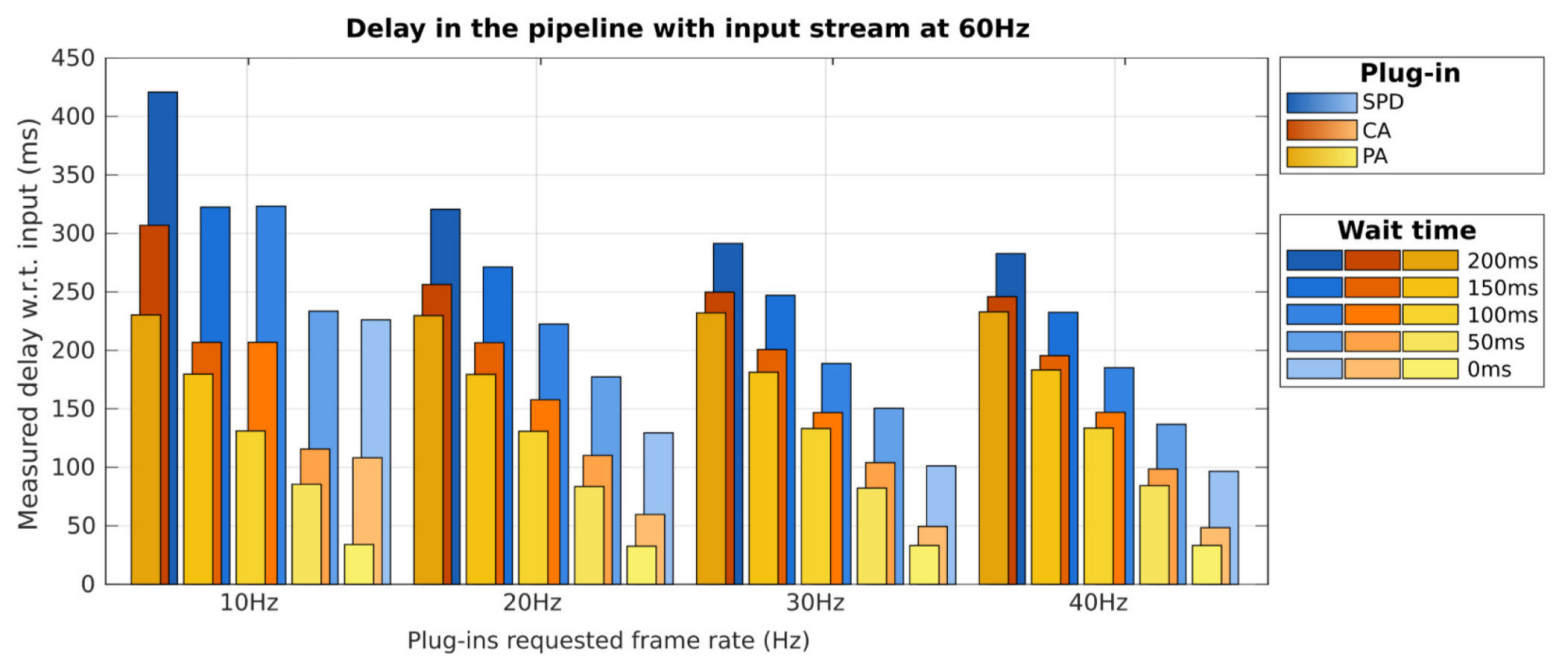
Measured delay between the output of each plug-in and real-time input stream when plug-ins are connected in sequence: Input → Python Algorithm (PA, in yellow) → Cpp Algorithm (CA, in orange) → SonoNet (SPD, in blue). The increasing wait time introduced in the PA plug-in is coded in the lighter to darker shade for each plug-in. When a higher frame-rate is requested, the delay decreases until around 30 Hz where other system parameters (e.g. memory, CPU, etc.) become the limiting factor. (For interpretation of the references to color in this figure legend, the reader is referred to the web version of this article.)

When the plug-ins are set up in sequence (accepting the input from the previous plug-in, configurations 2) to 5)), their individual execution time are not affected. As shown in Table 2 the decrease in execution time observed in the Standard Plane Detection plug-in as the wait time increases is due to more CPU resource available which is used in the pre-processing steps and other CPU based tasks. However each plug-in still needs to wait to receive the image from the previous plug-in, which introduces an added delay compared to processing images from the ‘Input’ stream. We measured the total delay between the output of a plug-in and the ‘Input’ stream by tracking the frame number from the input, and comparing the timestamp of that frame after processing. This delay will vary depending on the requested plug-in frame rate. Plug-ins check for the latest input frame at this requested frame rate (20 Hz by default), and new frames are dropped until the plug-in has finished the current processing task to avoid temporal drift. When idle, the next image is processed when the next periodic input check arrives. These two effects are illustrated in Fig. 3.

Because of the frame-dropping, the effective frame rate, i.e. the number of frames per second that each plug-in actually processes depends on three factors: (1) the execution time of the plug-in (which limits the maximum effective frame rate), (2) the execution time of the plug-ins that precede the current plug-in, if connected in sequence (the current plug-in will have to wait), and (3) other system parameters (e.g. memory, CPU, etc.). To illustrate the effect of these factors, we report the measured frame rates for each plug-in in Table 3. We also carried out a statistical significance test (N-way ANOVA) on the reported results.

As expected, in parallel (Par.), where all plug-ins use data from the Input stream, the CA and SPD plug-ins maintain the requested frame rate (20 Hz) independently of the other plug-ins. Obviously, the PA plug-in can only maintain the frame rate when no wait time is introduced, and then the frame rate decreases inversely to the wait time. This further demonstrates that two plug-ins, where the task is implemented in Python, can run independently in parallel. This is achieved by sharing the Python interpreter across plug-ins. In sequential execution (Seq.), the frame rate is limited by the wait time; as a result, the requested frame rate can only be achieved in certain cases, highlighted in light gray in the table (these cases are not statistically different to the required frame rate with *p* < 0.01). When requesting very high framerates, the pipeline might not be able to deliver at that framerate and the framerate will be capped to the maximum system framerate for that specific pipeline, which is around 26 Hz in this case (highlighted in dark gray in Table 3).

## 4 Impact

PRETUS will promote and facilitate real-time ultrasound imaging research for two main reasons: first, PRETUS is plug-in based, and plug-ins are self-contained in the sense that they implement the data processing, argument handling, user interface, image visualization, and any other display or input widgets; however, default modules and basic building blocks to develop plug-ins are provided in the Plugin library, and examples of plug-ins using both C++and Python are provided, simplifying the implementation of new plug-ins. Second, PRETUS implements user-transparent, multi-threaded plug-in execution with periodic calls to the tasks implemented by the plug-ins, to ensure that plug-ins always use the latest generated input image and that they run as close to real-time as possible. Crucially, a Python interpreter is shared across Python plug-ins enabling concurrent execution of independent, dynamically loaded Python plug-ins too.

Indeed, hundreds of research papers on ultrasound image analysis are published every year, most of which are trained and tested offline using an image database. As a result a platform to facilitate real-time data collection and implementing and evaluating computational methods in a realistic, real-time clinical scenario connected to an ultrasound imaging system is highly sought in US research. PRETUS allows the integration of C++and Python methods in a simple and flexible way, with minimal changes to an offline version that users and developers may already have. To this end, we have included tutorials on how to build plug-ins both in C++and Python. Conveniently, PRETUS also allows playing-back captured videos or sequences of images retrospectively and at acquisition frame rates, to replicate live sessions offline, in the lab. Being a lightweight and plug-in based software, PRETUS can accelerate translational research in ultrasound imaging for diagnostic and interventions.

Unlike other software, PRETUS implements real-time execution transparently to users and plug-in developers, by ensuring that, regardless of the performance and speed of the computational method, the latest input image will always be fed to the algorithm to avoid execution drift. PRETUS also ensures that plug-ins run in parallel and that their outputs and inputs can be interconnected at run time. Moreover, the way plug-ins are interconnected can be changed by the user at run time, or programmatically for example based on the output of an algorithm, making PRETUS extremely flexible. A unique feature of PRETUS is that plug-ins can be implemented in Python and in C++and multiple Python plug-ins can run concurrently by sharing the Python interpreter. Moreover, PRETUS is designed to connect to the video output of virtually any ultrasound system, so as to remove the impediment of manufacturer-specific formats and transmission protocols. However, a developer can easily implement a machine specific acquisition plug-in, using the provided input plug-ins as examples, if machine specific protocols are made available.

PRETUS also includes a file saving plug-in which turns the system into a powerful data collection software that can facilitate the acquisition of large amounts research data. Because multiple Input Streams can be captured simultaneously, synchronized multi-source data can also be captured and stored. The label or folder under which the images are saved can be changed in real time, allowing to easily annotate frame sequences (for example, by class) during the exam.

Overall, PRETUS has two main aims: first, facilitating research in real-time computational imaging, and specifically ultrasound imaging, by proving a lightweight application that can be easily extended and is designed to run in real time. And second, facilitating translation of computational methods by enabling easy integration of algorithms into a real-time framework, and providing a platform to test them in a live procedure or retrospectively using pre-recorded video data. We believe this will allow researcher to better show potential clinical impact of their computational methods in more realistic clinical situations.

## 5 Conclusions

We have discussed PRETUS, a plug-in based, real-time software for US imaging research. The software allows the collection of live ultrasound data and the use of algorithms (implemented in C++or Python). PRETUS loads plug-ins dynamically and a plug-in pipeline can be defined by the user at run time.

We have evaluated PRETUS with three examples, demonstrating real-time, concurrent execution of multiple plug-ins. PRETUS can be extended easily through more plug-ins and has the potential to enable researchers to evaluate their methods in real-time with minimal implementation efforts.

## Supplementary Material

MMC S1

MMC S2

MMC S3

Appendix

## Figures and Tables

**Table 1:** Overview of differences between PRETUS and two other widely used software that enable real-time imaging. In summary, PRETUS aims, by design, at being extremely lightweight and delegate all functionality to plug-ins that are loaded at run-time, and the plug-ins run asynchronously to ensure real-time behavior.

Software	Main app	Extensible in python?		Module execution
Slicer IGT	General purpose, fully featured	Yes	Python scripts can be loaded/ executed from main app	Asynchronous
MITK IGT	General purpose, fully featured	Yes	programmable python module	Asynchronous
PRETUS	Minimal, modular, all features come from plug-ins	Yes	Independent plug-ins that can run concurrently	Synchronous (user-defined frame-rate)

**Table 2:** Average ±standard deviation of the execution time, in ms, of each plug-in: Python Algorithm (PA), Cpp Algorithm (CA) and Standard plane detection (SPD), in configuration 1).

	Wait	0 ms	50 ms	100 ms	150 ms	200 ms
Time (ms)	PA	26.2 ± 5	76.7 ± 7	124.4 ± 3	173.6 ± 1	223.9 ± 1
	CA	0.9 ± 1	0.9 ± 1	0.8 ± 1	0.8 ± 0	0.7 ± 1
	SPD	17.6 ± 6	17.7 ± 9	15.0 ± 3	14.6 ± 1	14.8 ± 2

**Table 3:** Average ± standard deviation of the measured effective frame rate, in Hz, of each plug-in: Python Algorithm (PA), Cpp Algorithm (CA) and Standard plane detection (SPD), when the three plug-ins are executed in parallel (Par. row) or connected in sequence (Seq. rows) at the indicated user-requested frame rates (the same for all three plug-ins). Cells highlighted in light gray indicate that the measured and the requested frame rate match.

Conf.	Wait	0 ms	50 ms	100 ms	150 ms	200 ms
(1)	PA	20.0 ± 2.5	9.8 ± 1.0	6.6 ± 0.5	4.9 ± 0.3	3.9 ± 0.2
Par.	CA	20.0 ± 2.1	20.3 ± 3.4	20.4 ± 3.5	20.3 ± 3.7	20.3 ± 2.9
20 Hz	SPD	20.2 ± 3.1	19.9 ± 4.0	20.0 ± 4.4	20.0 ± 4.2	20.0 ± 3.3
(2)	PA	10.2 ± 1.5	9.6 ± 1.4	5.0 ± 0.1	5.0 ± 0.1	3.3 ± 0.6
Seq.	CA	10.0 ± 0.7	9.7 ± 1.4	5.0 ± 0.1	5.0 ± 0.1	3.3 ± 0.5
10 Hz	SPD	10.3 ± 1.9	9.8 ± 1.8	5.1 ± 0.6	5.0 ± 0.4	3.3 ± 0.6
(3)	PA	19.6 ± 2.6	9.9 ± 0.8	6.7 ± 0.3	5.0 ± 0.1	4.0 ± 0.1
Seq.	CA	19.6 ± 2.5	9.9 ± 0.8	6.7 ± 0.3	5.0 ± 0.1	4.0 ± 0.0
20 Hz	SPD	19.8 ± 3.2	10.0 ± 1.4	6.7 ± 0.5	5.0 ± 0.2	4.0 ± 0.1
(4)	PA	25.9 ± 5.0	10.6 ± 0.9	7.4 ± 0.5	5.2 ± 0.1	4.1 ± 0.6
Seq.	CA	26.0 ± 5.1	10.5 ± 0.9	7.4 ± 0.5	5.2 ± 0.1	4.1 ± 0.6
30 Hz	SPD	27.1 ± 7.9	10.8 ± 2.2	7.5 ± 1.1	5.2 ± 0.3	4.1 ± 0.6
(5)	PA	26.3 ± 4.0	11.4 ± 1.1	7.3 ± 0.4	5.4 ± 0.2	4.2 ± 0.1
Seq.	CA	26.4 ± 4.3	11.4 ± 1.2	7.3 ± 0.4	5.4 ± 0.2	4.2 ± 0.1
40 Hz	SPD	27.6 ± 7.9	11.7 ± 2.1	7.5 ± 1.7	5.4 ± 0.4	4.2 ± 0.2
