# Supplementary material for: PRETUS: A plug-in based platform for real-time ultrasound imaging research
Source: SoftwareX. Author manuscript; Available in PMC 2023 Jan 6. (PMC7614027; doi:10.1016/j.softx.2021.100959)
Supplement: Appendix [file EMS158114-supplement-Appendix.pdf]

open access, the author has applied a CC BY public copyright licence to any Author Accepted Manuscript version arising from this submission. This work was also supported by the Wellcome/EPSRC Centre for Medical Engineering, United Kingdom [WT203148/Z/16/Z] and by theNational Institute for Health Research (NIHR) Biomedical Research Centre, United Kingdom at Guy's andSt Thomas' NHS Foundation Trust and King's College London, United Kingdom. The views expressed are those of the author(s) and not necessarily those of the NHS, the NIHR or the Department of Health.

Appendix A. Plug-ins included with this release

A.1. File manager plug-in

This plug-in allows images to be read from a sub-directory hierarchy and transmits them through the pipeline at a certain frame-rate. Images can be 2D or 3D, and the mhd/raw format from the ITK library is preferred. Other formats supported by ITK can be also used by changing the expected file extension with the `-filemanager_extension` command line argument.

By default, images are transmitted in alphabetical order, therefore the file name will dictate the transmission order. Also, by default, images are transmitted at a constant frame rate of 20 images per second. A custom frame rate can be set by the user, in two ways: first, a constant frame rate between 0 and 200 can be set using the command line argument `-filemanager_framerate`. Second, if the mhd headers have the field `AcquisitionFrameRate`, then this value will be used, and can be different for each image. Additional options allow the last image to loop around when it is read or to ignore the header information.

This plug-in provides a widget that allows to scroll through the images rapidly and to play/pause the streaming. In pause mode, the same image keeps being transmitted at the default frame-rate, allowing the rest of the plug-ins to continue operating on the paused frame.

A.2. Video manager plug-in

This plug-in allows a video file to be read from the file system and transmits it through the pipeline. `Opencv` is used to read the video files so supported format depends on local configuration of opencv.

The video by default loops around when finished, but this can be disabled by the user using the command line argument `-videomanager_loop 0`. The video starts from the beginning by default, but an arbitrary start time can be set with `-videomanager_start_time <mm:ss>`. The video can also be played faster by setting a fast-forward factor with `-videomanager_ff <factor>`. This plug-in's widget enables interactively moving around in the video with a slider and to play/pause the streaming. In pause mode, the same video frame keeps being transmitted at the default frame-rate, allowing the rest of the plug-ins to continue operating on the paused frame.

A.3. Frame grabber plug-in

This plug-in allows a stream of images to be received in real-time from a video source, such as the video output of an ultrasound system, by using the Epiphone DVI2USB3.0 frame grabber (https://www.epiphan.com/products/dvi2usb-3-0/). The plug-in is currently implemented to convert the images to grayscale and pass it on to the rest of the pipeline as a single channel, 8 bit images.

A.4. Cpp algorithm plug-in

This plug-in performs a simple binary thresholding on the input image. The plug-in is conceived as a tutorial to illustrate how to develop C++plug-ins for PRETUS.

The Cpp Algorithm plug-in performs the thresholding operation using the ITK library. The threshold value can be set via command-line argument (`cppalgorithm_th <th>`) and edited in real-time using the slider in the plug-in's widget. An overlay of the input image and the thresholded image are shown on the plug-in's image widget.

A.5. Python algorithm plug-in

This plug-in performs a Gaussian blur on the input image. The plug-in is conceived as a tutorial to illustrate how to develop Python plug-ins for PRETUS.

The Python Algorithm plug-in performs the Gaussian blur operation using the SimpleITK Python library. The sigma value for the Gaussian kernel can be set via command-line argument (`pythonalgorithm_sigma <sigma>`) and edited in real time using the slider in the plug-in's widget. The blurred version of the input image is shown on the plug-in's image widget. The plug-in's worker waits a user-defined time (within the Python code) to simulate a longer task execution.

A.6. Standard plane detection (SonoNet)

This plug-in implements the fetal scan plane detection method described in [13]. The plug-in runs the method in every frame received from the input stream (which can be selected by the user).

The model makes a prediction about the scan plane corresponding to the image, and classifies the image into one of 13 standard views: '3VV' (cardiac three vessel view), '4CH' (cardiac four chamber), 'RVOT' (cardiac right ventricular outflow tract), 'LVOT' (cardiac left ventricular outflow tract), 'Abdominal', 'Brain (Cb.)' (cerebellum), 'Brain (Tv.)' (trans-ventricular), 'Femur', 'Kidneys', 'Lips', 'Profile', 'Spine (cor.)' (coronal), 'Spine (sag.)' (sagittal), or 'Background'. Illustrative examples of these views and their significance can be found in [18].

The algorithm yields a 13-element vector with a score indicating the probability of the image belonging to each class above. The plug-in packs this information into four fields in the output image header:

- "`Standardplanedetection_labels`", a string array with the original class labels in order.
- "`Standardplanedetection_confidences`", a float array with the probability for each class.
- "`Standardplanedetection_label`", a string with the label of the highest scoring class
- "`Standardplanedetection_confidence`", a float with the probability of the highest scoring class.

This output image is transmitted downstream the pipeline in the `Standardplanedetection` stream. The visualization widget displays these information as a bar plot with the classes and probabilities.

A.7. Image file writer plug-in

This plug-in allows images to be written to file, in real-time. The plug-in can write images from any stream, or multiple Streams, or all. Each received frame is written as a single image in mhd/raw format, which is well supported by imaging libraries

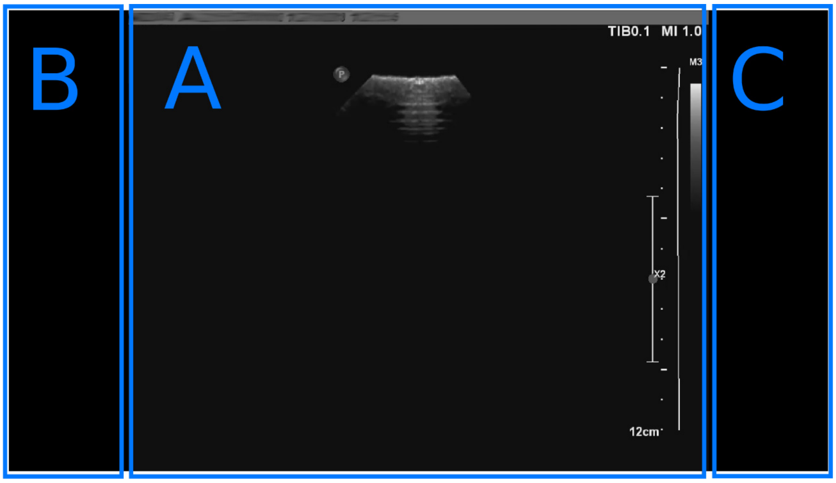

Fig. A.4. Interface of the visualization plug-in. The central frame (A) displays the images from a given stream in real time. The side frames (B and C) can be used to display widgets from individual plugins upstream the pipeline.

such as VTK and ITK and by imaging software such as Slicer and MITK.

This plug-in handles the header field "DO_NOT_WRITE" by not writing to file any image that has that key in the header, even if the image belongs to a stream that is being written. This allows other plug-ins to transmit images for visualization or for other plug-ins but not write them to file. For example, this is useful in the standard plane detection plug-in, where the user may not want to write the 'background' images to file, but still wants to visualize them in real time.

This plug-in implements a widget that shows the number of images that have been saved and allows to stop/resume the image saving via a checkbox.

A.8. GUI plug-in

The graphical user interface (GUI) plug-in is designed to display a stream of images and widgets around the images with information of the other plug-ins in the pipeline. The organization of the visualization window in shown in Fig. A.4.

All plug-ins can implement two types of widgets, declared in the `Plugin` parent class: plug-in widgets, that can be placed in panels B or C in the figure, and image widgets, that can be placed in panel A. By default, the GUI plug-in creates a colored frame around each widget that matches a colored frame around the image widget of the same plug-in (if available), as shown in Fig. 2. This can be disabled with the command line argument `--gui_usecolors 0`.

The GUI plug-in itself implements a widget (by default located in panel B) that allows to control the size of all image widgets.

Appendix B. Supplementary data

Supplementary material related to this article can be found online at https://doi.org/10.1016/j.softx.2021.100959.

References

[1] Che Chengqian, Mathai Tejas Sudharshan, Galeotti John. Ultrasound registration: A review. Methods 2017;115:128–43.
[2] Meiburger Kristen M, Acharya U Rajendra, Molinari Filippo. Automated localization and segmentation techniques for B-mode ultrasound images: A review. Comput Biol Med 2018;92:210–35.
[3] Liu Shengfeng, et al. Deep learning in medical ultrasound analysis: a review. Engineering 2019;5(2):261–75.
[4] Ungi Tamas, Lasso Andras, Fichtinger Gabor. Open-source platforms for navigated image-guided interventions. Med Image Anal 2016;33:181–6.
[5] Franz Alfred M, et al. Simplified development of image-guided therapy software with MITK-IGT. In: SPIE medical imaging 2012: image-guided procedures, robotic interventions, and modeling. 8316. International Society for Optics and Photonics; 2012.
[6] Tokuda Junichi, et al. OpenIGTLink: an open network protocol for image-guided therapy environment. Int J Med Robot Comput Assist Surgery 2009;5(4):423–34.
[7] Gomez Alberto, et al. Fast registration of 3D fetal ultrasound images using learned corresponding salient points. In: Fetal, infant and ophthalmic medical image analysis. Springer, Cham; 2017, p. 33–41.
[8] Gomez Alberto, et al. Image reconstruction in a manifold of image patches: Application to whole-fetus ultrasound imaging. In: International workshop on machine learning for medical image reconstruction. Springer, Cham; 2019.
[9] Zimmer Veronika A, et al. Towards whole placenta segmentation at late gestation using multi-view ultrasound images. In: International conference on medical image computing and computer-assisted intervention. Springer, Cham; 2019.
[10] Zimmer Veronika A, et al. A multi-task approach using positional information for ultrasound placenta segmentation. In: Medical ultrasound, and preterm, perinatal and paediatric image analysis. Springer, Cham; 2020, p. 264–73.
[11] Wright Robert, et al. LSTM spatial co-transformer networks for registration of 3D fetal US and MR brain images. In: Data driven treatment response assessment and preterm, perinatal, and paediatric image analysis. Springer, Cham; 2018, p. 149–59.
[12] Wright Robert, et al. Complete fetal head compounding from multi-view 3D ultrasound. In: International conference on medical image computing and computer-assisted intervention. Springer, Cham; 2019.
[13] Baumgartner Christian F, et al. Sononet: real-time detection and localisation of fetal standard scan planes in freehand ultrasound. IEEE Trans Med Imaging 2017;36(11):2204–15.
[14] Sinclair Matthew, et al. Human-level performance on automatic head biometrics in fetal ultrasound using fully convolutional neural networks. In: 2018 40th annual international conference of the IEEE engineering in medicine and biology society (EMBC). IEEE; 2018.
[15] Budd Samuel, et al. Confident head circumference measurement from ultrasound with real-time feedback for sonographers. In: International conference on medical image computing and computer-assisted intervention. Springer, Cham; 2019.
[16] Toussaint Nicolas, et al. Weakly supervised localisation for fetal ultrasound images. In: Deep learning in medical image analysis and multimodal learning for clinical decision support. Springer, Cham; 2018, p. 192–200.
[17] Kerdegari Hamideh, et al. Automatic detection of B-lines in lung ultrasound videos from severe dengue patients. In: IEEE international symposium in biomedical imaging. 2021.
[18] NHS Screening Programmes. Fetal anomaly screening: programme handbook. NHS Screen. Program; 2015.
